# Characterisation of cardiac health in the reduced uterine perfusion pressure model and a 3D cardiac spheroid model, of preeclampsia

**DOI:** 10.1186/s13293-021-00376-1

**Published:** 2021-04-20

**Authors:** Claire Richards, Kimberly Sesperez, Michael Chhor, Sahar Ghorbanpour, Claire Rennie, Clara Liu Chung Ming, Chris Evenhuis, Valentina Nikolic, Natasa Karadzov Orlic, Zeljko Mikovic, Milan Stefanovic, Zoran Cakic, Kristine McGrath, Carmine Gentile, Kristen Bubb, Lana McClements

**Affiliations:** 1 School of Life Sciences, Faculty of Science, University of Technology Sydney, Sydney, NSW Australia; 2 School of Biomedical Engineering, Faculty of Engineering and Information Technology, University of Technology Sydney, Sydney, NSW Australia; 3 The iThree Institute, University of Technology Sydney, Sydney, NSW Australia; 4 Department of Pharmacology and Toxicology & Department of Internal Medicine - Gynaecology, Medical Faculty, University of Nis, Nis, Serbia; 5 Department of Gynaecology and Obstetrics, Narodni Front, Belgrade, Serbia; 6 Medical Faculty, University of Belgrade, Belgrade, Serbia; 7 Department of Gynaecology and Obstetrics, Clinical Centre Nis, Nis, Serbia; 8 Department of Gynaecology and Obstetrics, General Hospital of Leskovac, Leskovac, Serbia; 9 The Kolling Institute, University of Sydney, Sydney, NSW Australia; 10 Biomedical Discovery Institute, Monash University, Melbourne, Australia

**Keywords:** Preeclampsia, Cardiovascular disease, Reduced uterine perfusion pressure, Cardiac spheroids, FKBPL

## Abstract

**Background:**

Preeclampsia is a dangerous cardiovascular disorder of pregnancy that leads to an increased risk of future cardiovascular and metabolic disorders. Much of the pathogenesis and mechanisms involved in cardiac health in preeclampsia are unknown. A novel anti-angiogenic protein, FKBPL, is emerging as having a potential role in both preeclampsia and cardiovascular disease (CVD). Therefore, in this study we aimed to characterise cardiac health and FKBPL regulation in the rat reduced uterine perfusion pressure (RUPP) and a 3D cardiac spheroid model of preeclampsia.

**Methods:**

The RUPP model was induced in pregnant rats and histological analysis performed on the heart, kidney, liver and placenta (*n* ≥ 6). Picrosirius red staining was performed to quantify collagen I and III deposition in rat hearts, placentae and livers as an indicator of fibrosis. RT-qPCR was used to determine changes in *Fkbpl*, *Icam1*, *Vcam1, Flt1* and *Vegfa* mRNA in hearts and/or placentae and ELISA to evaluate cardiac brain natriuretic peptide (BNP45) and FKBPL secretion. Immunofluorescent staining was also conducted to analyse the expression of cardiac FKBPL. Cardiac spheroids were generated using human cardiac fibroblasts and human coronary artery endothelial cells and treated with patient plasma from normotensive controls, early-onset preeclampsia (EOPE) and late-onset preeclampsia (LOPE); *n* = 3. FKBPL and CD31 expression was quantified by immunofluorescent labelling.

**Results:**

The RUPP procedure induced significant increases in blood pressure (*p* < 0.001), collagen deposition (*p* < 0.001) and cardiac BNP45 (*p* < 0.05). It also induced a significant increase in cardiac FKBPL mRNA (*p* < 0.05) and protein  expression  (*p* < 0.01). RUPP placentae also exhibited increased collagen deposition and decreased *Flt1* mRNA expression (*p* < 0.05). RUPP kidneys revealed an increase in average glomerular size (*p* < 0.05). Cardiac spheroids showed a significant increase in FKBPL expression when treated with LOPE plasma (*p* < 0.05) and a trend towards increased FKBPL expression following treatment with EOPE plasma (*p* = 0.06).

**Conclusions:**

The rat RUPP model induced cardiac, renal and placental features reflective of preeclampsia. FKBPL was increased in the hearts of RUPP rats and cardiac spheroids treated with plasma from women with preeclampsia, perhaps reflective of restricted angiogenesis and inflammation in this disorder. Elucidation of these novel FKBPL mechanisms in cardiac health in preeclampsia could be key in preventing future CVD.

**Supplementary Information:**

The online version contains supplementary material available at 10.1186/s13293-021-00376-1.

## Background

Preeclampsia is a dangerous cardiovascular disorder of pregnancy that affects around 5-8% of pregnancies and is one of the leading causes of maternal and foetal morbidity and mortality worldwide [1]. Preeclampsia typically presents during the second half of pregnancy (> 20 weeks of gestation) and is characterised by the new-onset of hypertension (> 140/90mmHg) in the presence of proteinuria (> 300mg/day) or other end-organ dysfunction, often that of the liver and kidneys [2, 3]. While low-dose aspirin has been investigated as a potential preventative treatment, the only cure for preeclampsia remains the delivery of the placenta and the baby, which is often at pre-term [4–6]. In addition to the immediate complications of a pregnancy affected by preeclampsia, women and babies affected by this disease are at a greater risk of developing post-partum cardiovascular, metabolic and neurological disorders [7–11]. In fact, women who are diagnosed with early-onset preeclampsia (EOPE; presenting prior to 34 weeks of gestation) are at a 9–10-fold increased risk of cardiovascular disease (CVD)-related deaths, while women diagnosed with late-onset preeclampsia (LOPE; presenting after 34 weeks gestation) are at a 2-fold increased risk of CVD-related death compared to those with normotensive pregnancies [12, 13]. Recently, several overlapping pathways and systems were identified between preeclampsia, hypertension and heart failure with preserved ejection fraction (HFpEF) including inflammation, angiogenesis, remodelling and haemostasis [14, 15].

Preeclampsia generally presents with two phenotypes: EOPE and LOPE; although term preeclampsia can also occur. While the delineation of these two phenotypes is still under investigation, EOPE often presents with a more severe disease state likely due to poor placentation and intrauterine growth restriction while LOPE results from poor maternal vascular adaptation perhaps due to underlying vascular dysfunction [16]. Even though the complex pathogenesis of this disease remains unknown, inappropriate placental development due to poor invasion and remodelling of maternal uterine vasculature and inability of the mother’s cardiovascular system to adapt to pregnancy-induced changes, appear to have critical roles. These factors result in aberrant angiogenesis, oxidative stress and inflammatory responses [17]. Anti-angiogenic factors including soluble fms-like tyrosine kinase-1 (sFlt-1) and soluble endoglin (sEng) are upregulated in preeclampsia and induce their effects by blocking the angiogenesis-promoting actions of vascular endothelial growth factor (VEGF) and placental growth factor (PlGF). Aberrant angiogenesis is also a key mechanism in diastolic dysfunction [18] and could perhaps be one of the lasting factors increasing the risk of CVD in women following preeclampsia.

A novel anti-angiogenic protein, FK506-binding protein like (FKBPL), has recently been identified as having predictive and diagnostic roles in preeclampsia [19] as well as being a determinant of CVD [20]. FKBPL is a divergent member of the immunophilin group that shares similar structure and functions to the FKBP members but lacks an essential residue in its peptidyl prolyl isomerases (PPIase) domain preventing its ability to exert this catalytic activity [21]. FKBPL has been demonstrated to have many functions including regulating glucocorticoid, androgen and oestrogen receptors as well as angiogenesis and stem cell differentiation [22–26]. It has been demonstrated that FKBPL induces anti-angiogenic effects by binding to the cluster of differentiation 44 (CD44) receptor on the surface of cells, regulating Notch or functioning as an Hsp90 co-chaperone [27, 28]. FKBPL has been recently implicated in endothelial function and key inflammatory pathways [19, 26]. While we have shown FKBPL to be more highly expressed in women with preeclampsia compared with healthy controls, its relationship and role in cardiovascular health in preeclampsia is unknown.

Animal models of preeclampsia have been difficult to establish, primarily due to the fact that preeclampsia can only be induced in most species other than humans and the presence of inter-species variations [29]. The most reliable animal model of preeclampsia is the surgically induced reduced uterine perfusion pressure (RUPP) model. This model has been shown to induce hypertension [30], proteinuria [30], renal dysfunction [30, 31], an anti-angiogenic state [32], inflammation [33–35], vasoconstriction [36], oxidative stress [37], cardiac dysfunction [38] and intrauterine growth restriction (IUGR) [39, 40] like that of preeclampsia in humans. Given the significant association between preeclampsia and CVD, a deeper investigation of the cardiac health in the RUPP model is required.

Furthermore, the use of an appropriate 3D cell culture model to study the effects of secreted placental factors on cardiac heart cells would enable a deeper understanding of the cardiac effects of preeclampsia. While traditional 2D cell culture methods are limited in their ability to recapitulate the cellular structure and function within a tissue, 3D cell culture methods with polymer-containing extracellular matrix (ECM), provide a 3D cell architecture allowing interaction in all spatial dimensions both with other cells and their environment. This allows for fine-tuning of the microenvironment by modifying properties including elasticity, stiffness, conductivity and porosity [41].

In this study, we demonstrate in the RUPP model of preeclampsia, the presence of diastolic dysfunction and cardiac fibrosis in association with increased cardiac FKBPL levels. We further show that cardiac spheroids containing cardiac fibroblasts and endothelial cells, treated with plasma from women with early- or late-onset preeclampsia, express higher protein levels of FKBPL reflective of early restricted angiogenesis.

## Methods

### Experimental animals

All animal experiments were approved by the Northern Sydney Local Health District Animal Ethics Committee at the Kolling Institute Building (Animal Ethics number: RESP/18/317). Pregnant Sprague-Dawley rats were fed a standard sterile chow diet and accessed water *ad libitum*. At gestational day (GD) 14, rats were randomised to the RUPP or Sham surgeries, which were performed as previously described [42, 43]. Briefly, silver clips were applied to the aorta above the iliac bifurcation (0.203mm ID) and both the right and left uterine arcades (0.100-mm ID) to reduce the blood flow to the uterus by ~ 40%. The sham procedure was performed by making a midline incision to open the lower abdominal cavity, though no clips were applied. On GD18, pregnant rats were anaesthetised with isoflurane (2–5% inhalation), and placed on a warm pad to maintain a body temperature of 37 °C before echocardiography was performed. They were then anaesthetised again in the afternoon, after 4 h of recovery, for catheter implantation and acute BP measurements. All rats were unconscious during BP recording but maintained at a similar and stable level of light anaesthesia (respiratory rates were controlled at similar levels). Rats were then euthanised immediately after blood pressure recording and post-mortem tissue collection performed. Blood, urine, organs, placentae and embryos were collected and stored in paraformaldehyde and liquid nitrogen. Hearts, placentae and embryos were also weighed before being processed.

### Echocardiography

Cardiac morphology and function were measured by echocardiography using a VisualSonics Vevo 3100 echocardiography system while rats were anaesthetised and body temperature maintained on a warm pad. Stroke volume (μl), cardiac output (mL/min), LV ejection fraction (%), fractional shortening (%), LV mass (mg) and LV anterior and posterior wall thicknesses at diastole (d) and systole (s) were measured from short-axis M-mode images. LV mass corrected (mg) was obtained by accounting for maternal body weight.

### Histology

Organs were formalin fixed, processed using Excelsior AS Tissue Processor (Thermo Fisher Scientific, USA), embedded in paraffin wax and stored at room temperature. Tissue sections (10μm) were subjected to haematoxylin and eosin (H&E) staining to visualise tissue morphology. A virtual slide was generated using a Zeiss Axioscan microscope and the size of 50 glomeruli per sample was measured using the polygon tool in Zen Lite 3.2 software to generate 2-dimensional surface area measurements. Overall cell number and cell size were measured in rat H&E stained liver sections using ImageJ. Picrosirius red staining was also performed to identify collagen I and III fibres as markers of fibrosis. Quantification of collagen deposition was performed using the colour threshold function of ImageJ.

### Immunofluorescent/immunohistochemical staining

Immunofluorescent staining of rat heart tissue was performed by de-waxing slides in xylene followed by rehydration through an ethanol series. Antigen retrieval was performed by heating in a pressure cooker with sodium citrate buffer (10 mM trisodium citrate, 0.05% Tween-20, pH 6.0) for 15 min. The slides were cooled in an ice bath and washed with distilled water twice. The slides were washed in PBST (phosphate buffer saline + 0.1% Tween-20), incubated in blocking buffer (1%BSA in PBST – PBS with 0.1% Triton-X) for 1 hour at room temperature and then incubated in a humidity chamber overnight with rabbit FKBPL polyclonal antibody (Proteintech, UK) diluted at 1:250 in blocking buffer. The following day, the slides were washed 3 times with PBST and incubated with donkey anti-rabbit AlexaFlour 488 (Abcam, UK) at 1:500 dilution with DAPI (Thermo Fisher, USA) in blocking buffer at room temperature in a humidity chamber. Six images per heart were taken at 40X magnification using an Olympus BX51 fluorescence microscope. ImageJ was used to calculate the mean greyscale value of the fluorescent intensity of FKBPL.

Rat liver sections were similarly dewaxed and rehydrated as above and stained for F4/80 by immunohistochemistry using a CSAII kit (Agilent, USA) according to the manufacturer’s protocols. Rabbit polyclonal F4/80 (Abcam, UK) was used at a 1:200 dilution and nuclei visualised by haematoxylin staining. A virtual slide was generated using a Zeiss Axioscan microscope and 3 random regions of interest were extracted and analysed by ImageJ to determine the average number and % area covered by F4/80 positive cells.

### Brain natriuretic peptide 45 enzyme-linked immunosorbent assay (ELISA)

BNP is released from the cardiac ventricles in response to diastolic and systolic dysfunction, which places additional stress on the heart walls [44, 45]. Rat cardiac lysates were used to measure BNP 45 concentrations in rat hearts using BNP 45 Rat ELISA Kit (Abcam, product #ab108816, UK) as per the manufacturer’s instructions. Optical density was detected using the Tecan infinite M200PRO, (Tecan Austria, GmbH) microplate reader.

### FKBPL ELISA

Specific FKBPL protein concentrations of rat hearts were measured by indirect ELISA developed in-house using a DuoSet® Ancillary Reagent Kit 1 (R&D Systems, USA). Briefly, PBS-diluted standards (FKBPL fusion protein; Proteintech, UK) and samples were added to each well of a 96-well plate (Immulon 1B, Flat bottom Microtiter plates, 3355). The plate was incubated overnight on a shaker at 4 °C. The following day, the plate was washed 3 times with 1x wash buffer before blocking buffer was added and incubated on a shaker for 2 h at room temperature. The plate was then washed 3 times with 1X wash buffer before primary antibody (Rabbit FKBPL Polyclonal antibody, Cat No. 10060-1-AP, 0.4 μg/ml in 1% BSA) was added to each well and incubated on a shaker for 2 h at room temperature. The plate was washed 3 times before the secondary antibody was applied (Anti-Rabbit: ab205722, 2 mg/ml, Dnk PAb to Rb IgG (HRP)), 100ng/ml in 1% BSA) followed by shaking for 2hr at room temperature. The plate was washed 5 times, and Colour Reagent A and Colour Reagent B were mixed in 1:1 and added to each well before incubating in the dark for 20 min at room temperature. Following incubation, stop solution was added and the absorbance measured at 450 nm and 540 nm using a Tecan infinite M200PRO, (Tecan Austria, GmbH) microplate reader. The concentration of unknown samples was calculated based on a Sigmoidal standard curve generated from the protein standards.

### Real-time quantitative reverse transcription polymerase chain reaction (RT-qPCR)

RT-qPCR was used to analyse mRNA expression of the genes *Fkbpl*, *intercellular adhesion molecule 1* (*Icam1)*, *vascular cell adhesion protein 1* (*Vcam1)*, fms-like tyrosine kinase 1 (*Flt1,* orvascular endothelial growth factor receptor 1; Vegfr1) and *vascular endothelial growth factor a (Vegfa)* in both the heart and/or placental tissue of the Sham and RUPP rats. RNA was extracted from the tissue by homogenising tissue sections with TRIsure reagent (Bioline, AU) and purified according to the manufacturer’s protocol. Extracted RNA was converted to cDNA by reverse transcription using Tetro cDNA synthesis kit (Bioline, AU) and corresponding primers for *Fkbpl*, *Icam1*, *Vcam1*, *Flt1* and *Vegfa* (Table 1). Real-time polymerase chain reaction (RT-PCR) was conducted using the prepared cDNA using a SensiFAST SYBR No-ROX Kit (Bioline, AU) according to the manufacturer’s protocol. The results were normalised to β-actin and transformed according to the ΔΔCT method.

**Table 1:** Nucleotide sequence of primers used in qPCR

Name of primers	Sequence (5′-3′)
β-actin (sense)	AAGACCTCTATGCCAACAC
β-actin (antisense)	TGATCTTCATGGTGCTAGG
Fkbpl (sense)	TGGCCTCTCAGGTCTGAACTA
Fkbpl (antisense)	TGGGGACTGCTGCTTAATCG
Icam1 (sense)	ATGTGCTATATGGTCCTCAC
Icam1 (antisense)	GTTTGACAGACTTCACCATC
Vcam1 (sense)	CTGATTATCCAAGGCTCTTC
Vcam1 (antisense)	CCATTAACAGACTTTAGCACC
Flt1 (sense)	CCAGAAGTCGTATGGTTAAAAG
Flt1 (antisense)	GCTGTGAGGTTTCTAAATAGC
Vegfa (sense)	GATAGAGTATATCTTCAAGCCG
Vegfa (antisense)	CTCATCTCTCCTATGTGCTG

### Generation of cardiac spheroids

Cardiac spheroids were generated by co-culturing human cardiac fibroblasts (HCFs) with human coronary artery endothelial cells (HCAECs) in hanging drop cultures at a ratio of 1:1 similar to the human heart, according to our previously established protocol [46]. Briefly, hanging drop cultures were generated using Perfecta 3D® 96-well hanging drop plates (3D Biomatrix, Ann Arbor, MI, USA) [47]. The spheroids were maintained with a fresh growth medium every 2 days and placed in a 37 °C incubator. Treatment of patient plasma serum was performed after spheroids were formed (3 days) [46]. Spheroids remained in incubation with plasma for 6 days. Cardiac spheroids were fixed using 1X PBS containing 4% paraformaldehyde, washed in PBS/0.01% sodium azide (PBSA), permeabilised using PBSA containing 0.02% Triton X-100, and blocked with 3% BSA in PBSA. Spheroids were incubated with appropriate primary (Anti-FKBPL rabbit pAb, Proteintech UK; Anti-CD31 mouse mAb, BD Biosciences, USA) and secondary (Anti-rabbit Donkey Cy3 conjugated, Abcam, UK; Anti-mouse Donkey Alexa-fluor 647 conjugated, Thermo Fisher, USA) antibodies, and nuclei were stained using Hoechst stain. Isotype IgG antibodies were used as negative controls.

#### Human plasma samples

Blood samples from women with early-onset (before 34 weeks’ gestation) or late-onset (after 34 weeks’ gestation) preeclampsia were collected following the diagnosis in EDTA coated tubes. Blood was centrifuged at 2000 rpm for 15 min at 4 °C to collect plasma. Preeclampsia was defined according to the 2013 ACOG guidelines by the clinicians in participating hospitals [48]. The study was approved by the local institutional review boards and written informed consent was obtained from all participants as per the principles outlined in the Declaration of Helsinki. The clinical characteristics of women with preeclampsia and normotensive controls are included in Table 3.

### Statistical analysis

Statistical analysis was performed in GraphPad Prism (v5). For analyses requiring comparison of the Sham and RUPP groups, an unpaired Student’s *t*-test was performed with statistically significant results equivalent to a *p* value of less than 0.05, where data was normalized (as per Shapiro-Wilk normality test), otherwise Mann-Whitney test was used; for *n* ≤ 4, a Mann-Whitney test was performed.

## Results

### RUPP procedure induces altered cardiovascular physiology in rats

On GD18, the sham and RUPP cohorts of rats were euthanised and organs collected and weighed. The scales used to weigh the organs of the first few animals were later determined to be insensitive and thus the results presented here are of the later samples (*n* = 5). At the end of study, RUPP rat hearts were significantly heavier than the controls (sham 1.02 ± 0.05 vs RUPP 1.22 ± 0.01, g; *n* ≥ 4, *p* = 0.008, Table 2); however, this did not translate to a significant difference between the wet heart: body weight ratio of each group (sham 0.30 ± 0.01 vs RUPP 0.32 ± 0.005, *n* ≥ 4; *p* = 0.3, Table 2).

**Table 2:** Maternal cardiac data in reduced uterine perfusion pressure rat model

	Sham (***n*** ≥ 6)	RUPP (***n*** ≥ 4)	***p*** value
Maternal body weight (before surgery), g	338.6 ± 17.5	379.5 ± 4.87	0.084
Maternal heart weight, g	1.02 ± 0.05	1.22 ± 0.01	0.008**
Heart: Body weight, %	0.304 ± 0.01	0.323 ± 0.005	0.303
Embryo resorption (%)	2.38 ± 1.51	11.22 ± 3.06	0.04*
Embryo weight, g	1.68 ± 0.025	1.61 ± 0.02	**0.04**
Placental weight, g	0.41 ± 0.009	0.39 ± 0.006	**0.047**
Heart rate, bpm	369.3 ± 4.8	398.8 ± 3.7	0.0006***
Systolic BP, mmHg	112.6 ± 1.3	127.8 ± 1.9	< 0.0001***
Diastolic BP, mmHg	87.6 ± 1.7	104.0 ± 1.8	< 0.0001***
MABP, mmHg	100.9 ± 1.5	116.7 ± 1.7	< 0.0001***
Stroke volume, μl	215.0 ± 4.326	230.0 ± 13.26	0.304
Cardiac output, mL/min	79 ± 3	85 ± 5	0.067
Ejection fraction, %	82 ± 2	78 ± 2	0.233
Fractional shortening, %	52 ± 2	49 ± 2	0.236
Corrected LV mass, mg	600 ± 14	706 ± 68	0.037*
LV anterior wall systolic, mm	2.9 ± 0.12	2.9 ± 0.11	0.672
LV anterior wall diastolic, mm	1.5 ± 0.04	1.8 ± 0.17	0.152
LV posterior wall systolic, mm	2.8 ± 0.14	2.9 ± 0.09	0.303
LV posterior wall diastolic, mm	1.6 ± 0.05	1.6 ± 0.11	0.994

*BP*, blood pressure; *LV*, left ventricular, *MABP*, mean arterial blood pressure; *RUPP*, reduced uterine perfusion pressure

Unpaired Student’s *t*-test or Mann-Whitney test depending on data distribution; *p < 0.05, ***p < 0.001

To assess whether the RUPP procedure was successful in inducing preeclampsia and associated cardiovascular changes in rats, blood pressure was measured on GD18 in both RUPP (*n* = 7) and sham surgery (*n* = 8) rats. Table 2 demonstrates that systolic blood pressure (sham 113 ± 1 vs RUPP 128 ± 2, mmHg; *p* < 0.001), diastolic blood pressure (sham 88 ± 2 vs RUPP 104 ± 2, mmHg; *p* < 0.001), mean arterial blood pressure (MABP) (sham 101 ± 1 vs RUPP 117 ± 2, mmHg; *p* < 0.001) and heart rate (sham 369 ± 5 vs RUPP 399 ± 4, bpm; *p* < 0.001) were all significantly increased in RUPP rats compared with sham control rats. We also observed intrauterine growth restriction (IUGR) measured by significantly reduced embryo weight (*p* = 0.04), placental weight (*p* = 0.047) and higher embryo resorption rate (*p* = 0.04; Table 2).

To investigate cardiac health in the RUPP model, echocardiography was performed. No statistically significant differences in stroke volume, cardiac output, ejection fraction or fractional shortening were observed between sham or RUPP rats (Table 2). The RUPP hearts had significantly higher corrected LV mass (sham 600 ± 14 vs RUPP 706 ± 68 mg, *p* = 0.037). No difference in the remaining echocardiography parameters was observed (Table 2).

### RUPP induces fibrosis and increased FKBPL expression in rat hearts

**Fig. 1:**
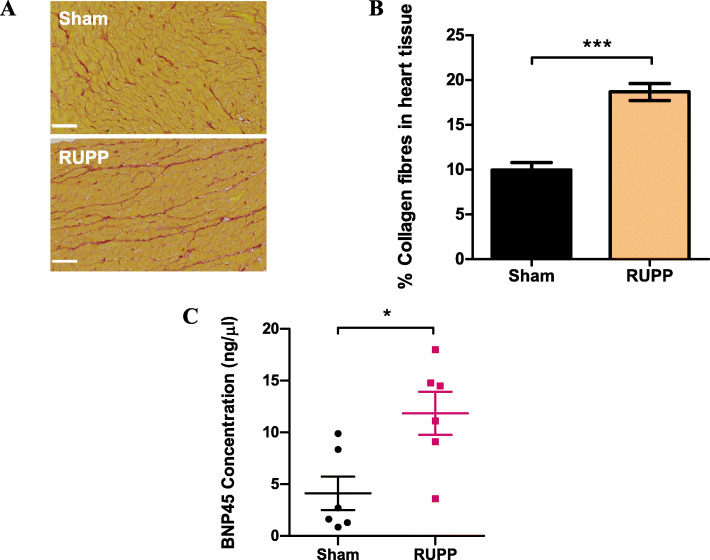
RUPP surgery increases collagen deposition and BNP45 concentration in rat hearts. **a**, **b** Paraffin-embedded rat hearts were sectioned at 10μm thickness and stained with picrosirius red to visualise collagen I/III (red) and muscle cell cytoplasm (yellow) in sham and RUPP rats. Scalebar = 50 μm. Images taken at × 5 using an Axioscan microscope were analysed for percentage area stained red to quantify collagen deposition as an indicator of cardiac fibrosis. **c** Rat heart protein isolated by homogenisation with RIPA lysis buffer was quantified by indirect ELISA against known standards to quantify protein concentration of BNP45. Data points are expressed as mean percentage ± SEM; *n* ≥ 6, unpaired Student’s *t*-test; *p < 0.05, ***p < 0.001

Picrosirius red staining of collagen I/III fibres revealed a statistically significant increase in collagen deposition in RUPP hearts compared with controls (Fig. 1a, b), indicating the development of cardiac fibrosis (sham 9.94 ± 0.8 vs RUPP 18.67 ± 0.9, %; *n* = 6, *p* < 0.001). Additionally, BNP, a well-established marker of cardiac hypertrophy and diastolic dysfunction [49], was analysed by ELISA using the rat heart protein lysates, showing a statistically significant increase in BNP protein concentration in RUPP hearts compared with sham controls (sham 4.12 ± 1.6 vs RUPP 11.84 ± 2.1, ng/μl, *n* = 6, *p* = 0.01, Fig. 1c).

**Fig. 2:**
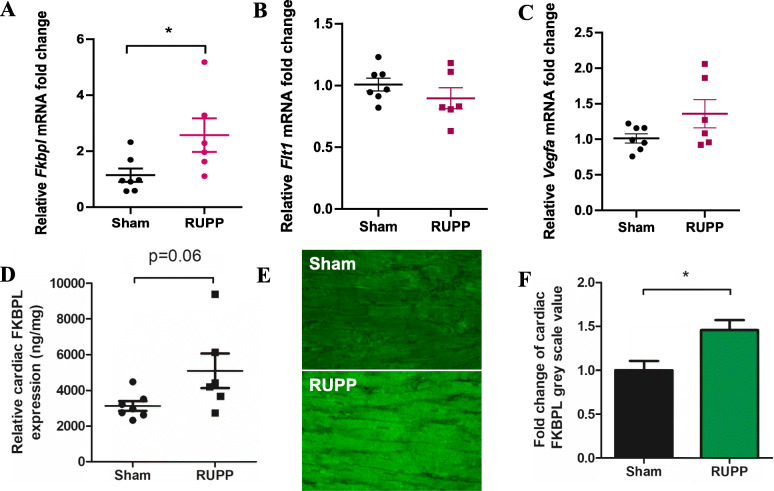
RUPP hearts express higher mRNA and protein levels of FKBPL. Total RNA extracted from rat hearts by TRIsure reagent was analysed by RT-qPCR to determine mRNA expression levels of *Fkbpl*, *Flt1* and *Vegfa*. Relative mRNA expression of **a** *Fkbpl*, **b** *Flt1* and **c** *Vegfa* mRNA levels comparing sham and RUPP hearts normalized to B-actin. Data presented as mean fold change ± SEM; *n* ≥ 6; Mann-Whitney *t*-test; *p < 0.05. **d** Fkbpl expression was verified at the protein level by enzyme-linked immunosorbent assay (ELISA), concentration calculated against a known standard and plotted against total mg of protein per sample. Data presented as mean ± SEM; *n* ≥ 6; unpaired Student’s *t*-test. **e**, **f** Cardiac FKBPL expression was further analysed by immunofluorescent staining and intensity measured using ImageJ by analysing greyscale value in six × 40 images per animal. Image brightness was adjusted equally for representative images displayed. Data plotted as mean fold change ± SEM; *n* ≥ 5; unpaired Student’s *t*-test; *p < 0.05

RT-qPCR showed a significant increase in the mRNA expression of *Fkbpl* in RUPP rat hearts (sham 1.14 ± 0.24 vs RUPP 2.57 ± 0.6, fold change, *n* = 6, *p* = 0.03, Fig. 2a). However, there were no statistically significant differences in the mRNA expression of *Flt1* (sham 1.008 ± 0.052 vs RUPP 0.8972 ± 0.085, *n* ≥ 6, *p* > 0.05, Fig. 2b) or *Vegfa* (sham 1.014 ± 0.065 vs RUPP 1.360 ± 0.198, *n* ≥ 6, *p* > 0.05, Fig. 2) between the hearts of RUPP and sham rats. Similarly, no significant differences between the mRNA expression of *Icam1* or *Vcam1* in the hearts of sham or RUPP rats (Additional File 1, Supplementary Figure 1). Given that FKBPL protein is prone to post-translational modifications, the difference in mRNA expression needed to be confirmed at the protein level. Indirect ELISA revealed a trend towards an increase in FKBPL protein concentration in RUPP hearts compared with Sham controls (sham 3123 ± 270.3 vs RUPP 5095 ± 969.2, ng/mg, *n* ≥ 6, *p* = 0.059, Fig. 2d). This trend was supported by a statistically significant increase in FKBPL expression observed between the RUPP heart tissue compared with controls (sham 1.0 ± 0.1 vs RUPP 1.46 ± 0.1, *n* = 5, *p* = 0.018, Fig. 2e, f).

### RUPP procedure leads to fibrosis and decreased Flt1 expression in rat placentae

**Fig. 3:**
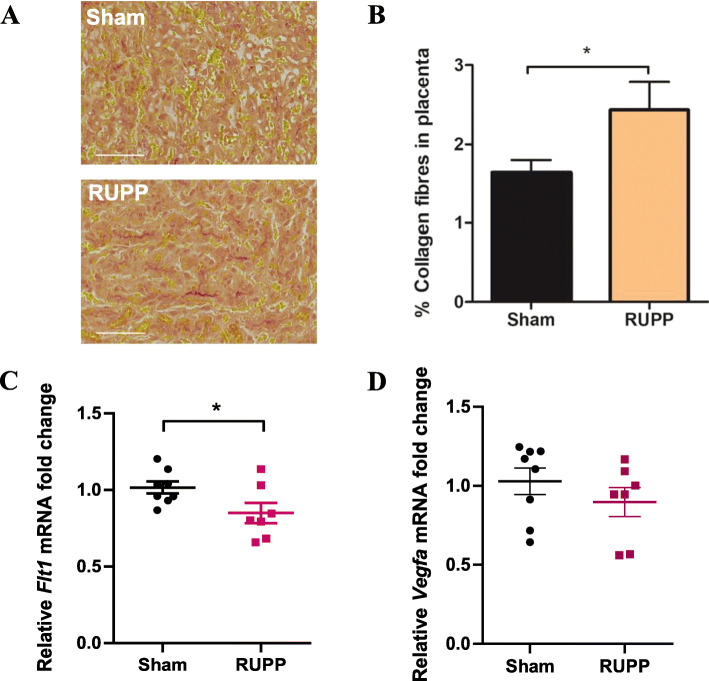
RUPP rats have increased collagen deposition and decreased *Flt1* mRNA expression in their placentae. **a** Paraffin-embedded rat placentae were sectioned at 10μm thickness and stained with picrosirius red to visualise collagen I and III fibres (red) in sham and RUPP hearts. Scalebar = 100μm. **b** Images of picrosirius red-stained tissue were taken at × 5 using an Axioscan microscope and analysed for percentage of area stained red to quantify collagen deposition as an indicator of placental fibrosis. Data points are mean ± SEM; *n* = 7, unpaired Student’s *t*-test; *p < 0.05. RNA extracted from rat placentae by TRIsure reagent was analysed by RT-qPCR to determine mRNA expression levels of **c** *Flt1* and **d** *Vegfa*. Data presented as mean fold change ± SEM; *n* = 7; Mann-Whitney *t*-test; *p < 0.05

Picrosirius red staining of collagen fibres I and III revealed a statistically significant increase in collagen deposition in RUPP placentae, indicating the presence of some level of placental fibrosis (sham 1.6 ± 0.16 vs RUPP 2.4 ± 0.35, %, *n* = 7, *p* = 0.03; Fig 3a, b).

Similar to the rat hearts, total RNA was isolated and analysed by RT-qPCR to determine any changes to mRNA expression levels of *Icam1*, *Vcam1*, *Flt1* and *Vegfa*. As shown in Supplementary Figure 2, there was no statistically significant difference in expression of *Icam1* or *Vcam1* mRNA between the sham or RUPP groups*.* There was, however, a decrease in expression of *Flt1* mRNA in RUPP placentae compared with sham controls (sham 1.016 ± 0.04 vs RUPP 0.85 ± 0.07, fold change, *n* = 7, *p* = 0.048, Fig. 3c). There was no significant difference in the *Vegfa* mRNA levels in placentae between the Sham and RUPP rats (sham 1.029 ± 0.085 vs RUPP 0.897 ± 0.091, *n* = 7, *p* = 0.189, Fig. 3d).

### Increased glomerular size is observed in RUPP kidneys

**Fig. 4:**
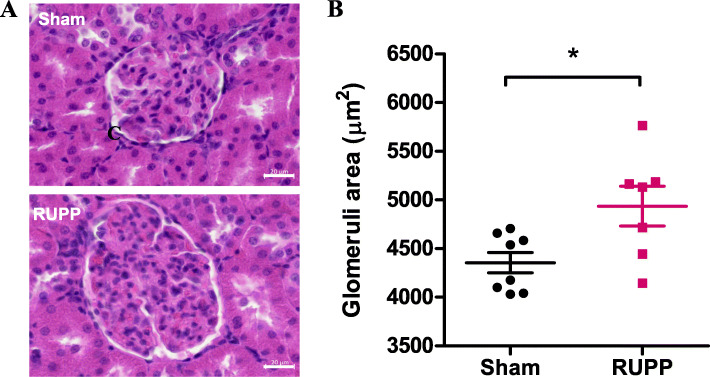
RUPP rats present with larger glomeruli in rat kidneys. Paraffin-embedded rat kidneys were cut at 10-μm sections and stained with H&E staining to visualise tissue morphology. **a** Images of entire tissue sections were taken at × 4, × 10 and × 20 objective using an Axioscan microscope to produce virtual slides of sham and RUPP kidneys. Scalebar = 20 μm. **b** Average glomerular area was measured at a × 20 magnification using ZEN Lite 3.2 software. Data plotted as mean ± SEM; *n* = 7; unpaired Student’s *t*-test; *p < 0.05

H&E staining of rat kidneys was performed to visualise tissue morphology. The area of 50 glomeruli within kidney sections was measured per animal using Zen Lite (v3.2). The mean of these results demonstrated a significant increase in the overall size of the glomeruli within the Bowman’s capsules’ of RUPP rats compared with sham controls (sham 4355 ± 103.5 vs RUPP 4935 ± 204.6, μm^2^, *n* = 7, *p* = 0.03; Fig. 4). While the characteristic endothelial swelling was not observed, this data suggests glomerular endotheliosis may be present in RUPP rats, which has previously been observed in an sFlt-1 administration model of preeclampsia in rats [50].

### Signs of potential liver inflammation are observed in RUPP livers

Given preeclampsia often manifests in liver dysfunction, we performed histological analysis of rat livers. H&E analysis of rat liver tissue (presented in Supplementary Figure 3) revealed an increase in the population of small cells (5–15 μm^2^) in RUPP rats. To further decipher the type of immune cells present in the liver, we performed immunohistochemistry staining for F4/80, a marker of macrophages, given their key role in maintaining liver health. Supplementary Figure 3 e–g shows that there was a trend towards an increased number of macrophages in the liver of RUPP rats compared with controls, though this was not significant. Further characterisation of these cell populations by immunohistochemistry would enable their identification and determine whether this change is indicative of liver inflammation. Picrosirius red staining revealed no significant increase in collagen I or III fibres (Supplementary Figure 4).

### Plasma samples from women with preeclampsia lead to increased FKBPL expression in cardiac spheroids

**Fig. 5:**
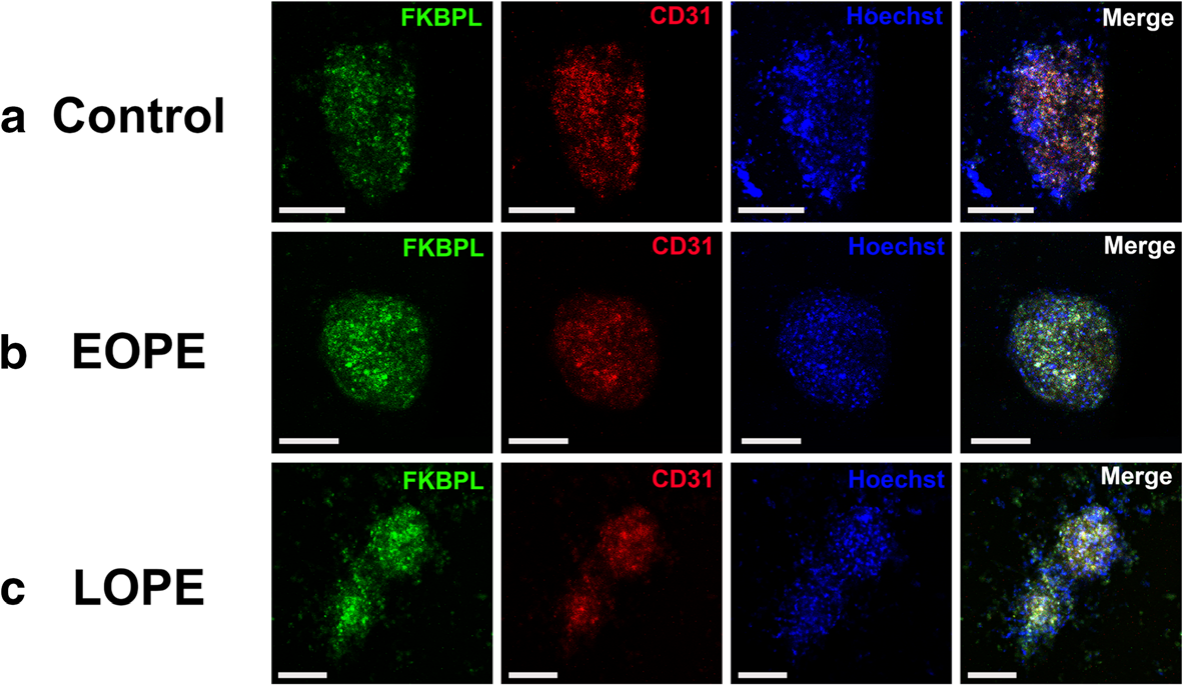
Immunofluorescent images of cardiac spheroids treated with plasma samples from healthy control, early-onset and late-onset preeclampsia patients. Cardiac spheroids were generated in hanging droplets by co-culturing primary human cardiac fibroblast cells (HCFs) and human coronary artery endothelial cells (HCAECs) in a 1:1 ratio. Following formation of 3D structures, the spheroids were treated with human plasma samples from women with or without preeclampsia. After plasma treatment, spheroids were fixated and permeabilised prior to labelling with antibodies (FKBPL, CD31) and fluorescent stain (Hoechst). **a** Spheroids treated with plasma from normotensive control pregnancies. **b** Spheroids treated early-onset preeclampsia (EOPE) patient plasma. **c** Spheroids treated with late-onset preeclampsia (LOPE) patient plasma

**Fig. 6:**
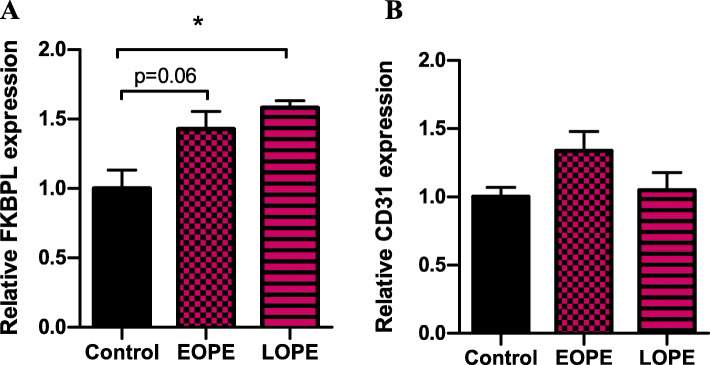
Expression of FKBPL and CD31 in cardiac spheroids treated with plasma from healthy control, early-onset and late-onset preeclampsia patients. Cardiac spheroids generated by co-culturing primary human cardiac fibroblast cells (HCFs) and human coronary artery endothelial cells (HCAECs) were treated with patient plasma from normotensive controls, early-onset preeclampsia and late-onset preeclampsia. Immunofluorescent expression of **a** FKBPL and **b** CD31 in cardiac spheroids was quantified. Data plotted as mean ± SEM; *n* = 3; ordinary one-way ANOVA with Tukey’s multiple comparison test; *p < 0.05

Human 3D cardiac spheroids were generated by co-culturing cardiac fibroblasts and endothelial cells to analyse FKBPL and CD31 protein expression following treatment with normotensive (healthy), EOPE and LOPE human plasma samples. Generation of cardiac spheroids containing only cardiac fibroblasts and endothelial cells in the absence of myocytes was based on our previous study, that highlighted the role played by fibroblasts and their extracellular matrix on endothelial cell network formation, a critical aspect of modelling mechanisms regulating new blood vessel formation in vitro [46]. Following incubation in human plasma from women with EOPE, LOPE and normotensive controls, cardiac spheroids were fixed and probed for immunofluorescent visualisation of FKBPL and CD31 proteins (Fig. 5). Semi-quantitative protein expression was measured by quantifying the immunofluorescent intensity of stained spheroids. Our analysis demonstrated a trend towards increased FKBPL expression in EOPE-treated spheroids compared with normotensive control plasma (control 1.00 ± 0.13 vs EOPE 1.43 ± 0.125, *n* = 3, *p* = 0.06, Fig. 6a) and a significant increase in the expression of FKBPL in LOPE-treated spheroids compared with normotensive control plasma (control 1.00 ± 0.13 vs LOPE 1.58 ± 0.05; *p* = 0.03; Fig. 6a). There was also a trend towards an increase in CD31 expression (used to measure endothelial cell network formation in cardiac spheroids) in EOPE-treated spheroids compared with control. However, this trend was not statistically significant (control 1.00 ± 0.07 vs EOPE 1.34 ± 0.14, *n* = 3, *p* = 0.18, Fig. 6b). There was no significant difference in CD31 protein expression between LOPE-treated spheroids compared with healthy control (control 1.00 ± 0.07 vs LOPE 1.05 ± 0.129; *p* = 0.95; Fig. 6b) after treatment with patient plasma samples. No significance was determined between EOPE- and LOPE-treated cardiac spheroids in terms of FKBPL or CD31 expression. There was no difference in age, BMI and parity between the groups whereas gestational age at sampling was significantly lower as well as blood pressure in women with preeclampsia (Table 3).

**Table 3:** Clinical characteristics of women with preeclampsia and normotensive controls

	Controls (***n*** = 3)	EOPE (***n*** = 3)	LOPE (***n*** = 3)	***p*** values
Age (years)	32 ± 5.6	32 ± 8.4	33 ± 3.1	0.94
BMI (kg/m^2^)	27 ± 6	31.3 ± 4.6	27.4 ± 1.3	0.47
Gestational age (weeks)	39.3 ± 0.6	31.5 ± 0.9	35.5 ± 1.8	**< 0.001**
SBP (mmHg)	116.7 ± 5.8	164.3 ± 14	163.3 ± 2.9	**< 0.001**
DBP (mmHg)	76.7 ± 5.8	112.3 ± 6.8	106.7 ± 5.8	**< 0.001**
Number of pregnancies	1.7 ± 0.6	2 ± 1.7	1 ± 0	0.53

*BMI*, body mass index; *SBP*, systolic blood pressure; *DBP*, diastolic blood pressure. Statistical analysis

## Discussion

In this study, surgically reduced perfusion to the uterus in pregnant rats resulted in anatomical and physiological alterations to maternal hearts, placentae and kidneys with a number of features of preeclampsia being demonstrated. The cardiovascular dysfunction particularly in terms of heart health has not been extensively studied in this model of preeclampsia before [51]. Given the well-established association between preeclampsia and increased risk of developing future CVD [52], this is an important aspect in modelling the manifestation of preeclampsia. In fact, recently published results describe the overlapping mechanisms between preeclampsia and future cardiovascular disease with angiogenesis- and inflammatory-related pathways playing a key role [15].

The RUPP model is one of the most reliable in vivo models for studying preeclampsia. Although there is substantial evidence published exploring this model’s reflection of human features of preeclampsia, we aimed to confirm the cardiovascular phenotype in RUPP rats and to determine the regulation of an emerging anti-angiogenic protein, FKBPL, which also regulates some key inflammatory pathways [26, 53]. This is the first study that explores the cardiovascular health in the RUPP model of preeclampsia and the association of FKBPL in cardiac dysfunction in preeclampsia.

A significant increase in systolic, diastolic and mean arterial blood pressure confirmed that the RUPP procedure had been successful and increased heart rate with LV hypertrophy was evident, as determined by echocardiography. This is in line with what was demonstrated before in the RUPP model [54, 55]. Additionally, signs of IUGR were observed with a significant decrease in foetal and placental weight following reduced placental perfusion, as has previously been described [54]. It is difficult to determine whether this model is more representative of EOPE or LOPE. LOPE is diagnosed from 34 weeks of gestation, it is a less understood phenotype of preeclampsia, and it likely occurs secondary to maternal microvascular diseases, reflective of underlying vascular dysfunction. It seems to develop due to maternal inability to meet metabolic and cardiovascular demands of the growing foetus. On the other hand, EOPE is usually diagnosed before 34 weeks of gestation and it is associated with foetal growth restriction as well as inadequate or incomplete trophoblast invasion of SUAs, often implicating placenta as the root cause [17]. Our analysis of placental collagen deposition found RUPP placenta to be more fibrotic compared with sham controls.

Picrosirius red staining data of RUPP hearts suggests the presence of cardiac fibrosis compared to sham controls, which is consistent with the preeclampsia phenotype and increased risk of CVD including cardiac fibrosis, later in life [56–58]. These results are also consistent with another study characterising the cardiac effects of RUPP in rats that showed a significant increase in collagen I and III fibrotic markers in RUPP hearts [59], although these effects have been shown to be reversed following a RUPP pregnancy [55]. This cardiac remodelling and fibrosis is likely driven by inflammation, as shown in published literature [60], and inflammation is a common feature of both EOPE and LOPE [61]. We have noted a significant increase in cardiac levels of BNP, which is indicative of diastolic dysfunction and cardiac hypertrophy that is supported by other studies’ findings in preeclampsia models [59, 62].

In addition to cardiac fibrosis, FKBPL was significantly increased at the mRNA level as a result of the RUPP procedure. However, FKBPL is a chaperone protein and prone to post-transcriptional modifications so quantification of its expression at the protein level is more informative [63]. Therefore, ELISA was performed on rat heart protein lysates and immunofluorescent staining of sectioned tissue, which indeed confirmed an overall increase in FKBPL protein levels in RUPP hearts. This is aligned with the positive correlation observed previously between FKBPL plasma levels and diastolic dysfunction or BNP plasma levels in human samples [20]. Recent studies show that FKBPL regulates inflammatory STAT3 signalling, via CD44 and NFkB, which are both implicated in preeclampsia [26, 53, 64, 65]. This data supports our hypothesis that FKBPL may be a novel mechanism of cardiovascular damage in preeclampsia. Recent data from our group has shown that FKBPL is increased as a result of inflammation through TGF-β stimulation in cardiac fibroblasts [66]. FKBPL is also increased in plasma and placentae from women with preeclampsia [19].

Histological analysis of picrosirius red staining in rat placentae revealed a significant increase in collagen I and III deposition in RUPP samples, potentially indicating the presence of placental fibrosis. Placental fibrosis has been found to occur in women with preeclampsia compared with normotensive controls [67]. Additional quantification of fibrotic factors including connective tissue growth factor (CTGF) or fibronectin in these placentae could aid in determining the level of fibrosis. Of note, placental fibrosis is a prominent feature of preeclamptic placentae and has been shown to be associated with the activation of stromal fibroblasts via the TGF-β1 signalling pathway [67]. Reduced angiogenesis as depicted by decreased *Flt1* expression further supports this finding.

Given that preeclampsia is frequently associated with altered renal function and histology, RUPP and sham kidneys were inspected for altered tissue morphology showing that glomeruli in RUPP samples had a significantly larger surface area. While the RUPP model has not yet been described as displaying glomerular endotheliosis, a characteristic feature of preeclampsia [68, 69], some changes to kidney tissue were previously noted. While this measurement is limited to 2-dimensional analysis rather than a 3-dimensional measurement of each glomerular structure, it does suggest that there may be early signs of glomerular endotheliosis. Electron microscopy or glomerular filtration rate would be important tools to measure this in future experiments. Moreover, previous studies have demonstrated an increase in proteinuria and a decrease in glomerular filtration rate following the RUPP procedure, with the latter lasting up to 8 weeks postpartum [54, 55]. We also observed the signs of liver inflammation; however, this needs to be characterised further to determine the content of inflammatory immune cells.

The effects of plasma secretome collected from women with preeclampsia on FKBPL expression levels were evaluated in in vitro 3D cardiac spheroid model to determine its potential role in the human heart. CD31 as a marker of endothelial cells was used to evaluate a potential correlation between FKBPL expression and endothelial cell network formation within cardiac spheroids. Our analysis determined that FKBPL expression was elevated in LOPE-treated spheroids when compared with healthy controls. A similar trend was measured in EOPE-treated spheroids compared with healthy controls, while this was not statistically significant. Therefore, it appears that circulating factors, likely inflammatory, in maternal plasma during preeclampsia may induce an overexpression of FKBPL in cardiac fibroblast and/or endothelial cells. While we did not observe a significant change in CD31 expression, potentially due to the short course of our experiments, future studies using our model for longer times may help to further evaluate the mechanisms regulating the role of FKBPL on endothelial cell network formation. We have previously shown that cardiac spheroids can model cardiac-specific pathophysiological conditions in a time-dependent manner. For instance, cardiac fibrosis is only measurable in cardiac spheroids after 3 days of treatment with TGF-β1, while doxorubicin-mediated toxic effects can be detected after 24 days in culture [46, 70]. While this study reports the initial response of human heart tissues to preeclampsia-derived plasma, future studies may help to further evaluate the underlying mechanisms that regulate the effects of preeclamptic plasma on endothelial cell network formation.

## Perspectives and significance

In our RUPP model, cardiac dysfunction including diastolic dysfunction and cardiac fibrosis was observed as a result of preeclampsia. Furthermore, we demonstrate, for the first time, that FKBPL is upregulated in the hearts of RUPP rats and cardiac spheroids treated with plasma from women with preeclampsia, supporting our hypothesis that this novel anti-angiogenic mechanism is associated with cardiovascular dysfunction in preeclampsia.

## Supplementary Information

**Additional file 1: Additional Figure** **1****.** Relative mRNA expression levels of endothelial dysfunction markers Icam1 and Vcam1 in RUPP hearts. Total RNA was collected from 30-50mg sections of cryopreserved rat hearts by a TRIsure reagent protocol. The relative levels of (A) Icam1 and (B) Vcam1 mRNA were quantified by real-time polymerase chain reaction (RT-PCR) adjusted to β-actin. Data presented as mean fold change ± SEM; n=7; unpaired t-test. **Additional Figure** **2****.** Relative mRNA expression levels of endothelial dysfunction markers *Icam1* and *Vcam1* in RUPP placentae. Total RNA was collected from 30-50mg sections of cryopreserved rat hearts by a TRIsure reagent protocol. The relative levels of (A) *Icam1 and* (B) *Vcam1* mRNA were quantified by real-time polymerase chain reaction (RT-PCR) adjusted to β-actin. Data presented as mean fold change ± SEM; n=7; unpaired t-test. **Additional Figure** **3****.** Cell size and count of RUPP livers and F4/80 quantification by IHC. Formalin-fixed, paraffin-embedded rat liver tissue was sectioned at 10μm thickness and stained with H&E to visualise tissue morphology. Images of entire tissue sections were taken using an Axioscan microscope to produce virtual slides of Sham and RUPP livers. (A) Number of cells and (B) cell size of liver tissue were measured and compared. H&E rat livers were further analysed by ImageJ to detect and count cells with area 5-15μm2 (indicated by arrows) in (C) Sham and RUPP livers. Scale bar = 50μm. (D) Number of small cells in livers plotted as mean ± SEM, n=7; unpaired student’s t-test; *<0.05, **<0.01. (E) Liver sections were further stained by immunohistochemistry with F4/80 antibody to detect macrophages. The (F) number and (G) % Area of F4/80-positive cells were analysed by ImageJ and plotted as mean ± SEM, n≥6; unpaired student’s t-test. **Additional Figure** **4****.** Degree of fibrosis of RUPP livers as determined by picrosirius red staining of collagen fibres. FFPE rat livers were sectioned at 10μm thickness and stained with picrosirius red to reveal collagen I and III fibres (red) in (A) sham and (B) RUPP hearts. Scalebar = 100μm. Images taken at 5x using an Axioscan microscope were analysed for percentage area stained red to quantify collagen deposition as an indicator of fibrosis (C). Data points are mean ± SEM; n=7, unpaired student’s t-test.

## Data Availability

The datasets generated and analysed in the current project are available from the corresponding author upon reasonable request.
