# Supplementary material for: Characterisation of cardiac health in the reduced uterine perfusion pressure model and a 3D cardiac spheroid model, of preeclampsia
Source: Biol Sex Differ. 2021 Apr 20;12:31. doi: 10.1186/s13293-021-00376-1 (PMC8056582; doi:10.1186/s13293-021-00376-1)
Supplement: Supplementary file 1 — Additional file 1: Additional Figure 1. Relative mRNA expression levels of endothelial dysfunction markers Icam1 and Vcam1 in RUPP hearts. Total RNA was collected from 30-50mg sections of cryopreserved rat hearts by a TRIsure reagent protocol. The relative levels of (A) Icam1 and (B) Vcam1 mRNA were quantified by real-time polymerase chain reaction (RT-PCR) adjusted to β-actin. Data presented as mean fold change ± SEM; n=7; unpaired t-test. Additional Figure 2. Relative mRNA expression levels of endothelial dysfunction markers Icam1 and Vcam1 in RUPP placentae. Total RNA was collected from 30-50mg sections of cryopreserved rat hearts by a TRIsure reagent protocol. The relative levels of (A) Icam1 and (B) Vcam1 mRNA were quantified by real-time polymerase chain reaction (RT-PCR) adjusted to β-actin. Data presented as mean fold change ± SEM; n=7; unpaired t-test. Additional Figure 3. Cell size and count of RUPP livers and F4/80 quantification by IHC. Formalin-fixed, paraffin-embedded rat liver tissue was sectioned at 10μm thickness and stained with H&E to visualise tissue morphology. Images of entire tissue sections were taken using an Axioscan microscope to produce virtual slides of Sham and RUPP livers. (A) Number of cells and (B) cell size of liver tissue were measured and compared. H&E rat livers were further analysed by ImageJ to detect and count cells with area 5-15μm2 (indicated by arrows) in (C) Sham and RUPP livers. Scale bar = 50μm. (D) Number of small cells in livers plotted as mean ± SEM, n=7; unpaired student’s t-test; *<0.05, **<0.01. (E) Liver sections were further stained by immunohistochemistry with F4/80 antibody to detect macrophages. The (F) number and (G) % Area of F4/80-positive cells were analysed by ImageJ and plotted as mean ± SEM, n≥6; unpaired student’s t-test. Additional Figure 4. Degree of fibrosis of RUPP livers as determined by picrosirius red staining of collagen fibres. FFPE rat livers were sectioned at 10μm thickness and stained with [file 13293_2021_376_MOESM1_ESM.pptx]

## Slide 1
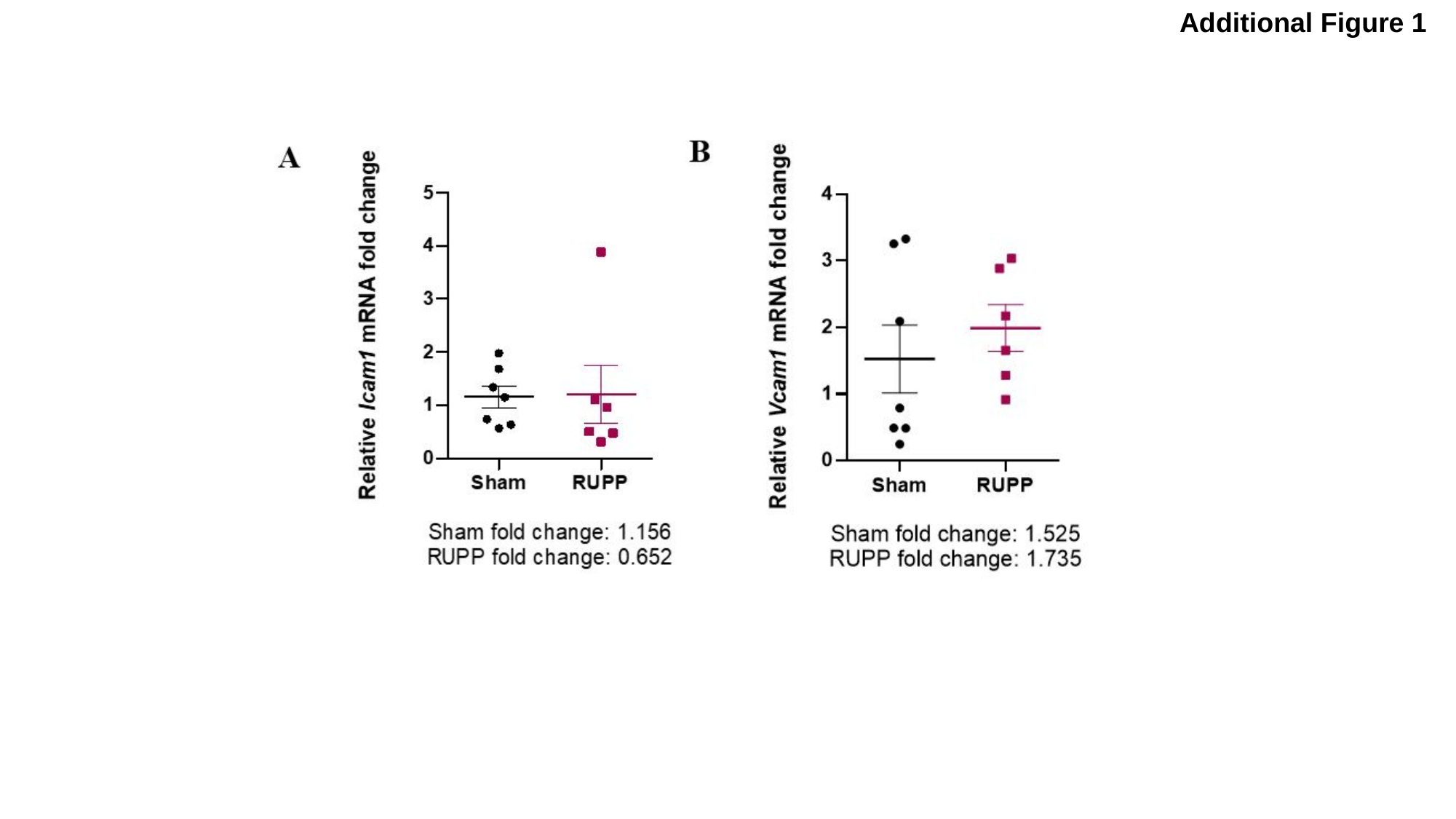

Additional Figure 1

## Slide 2
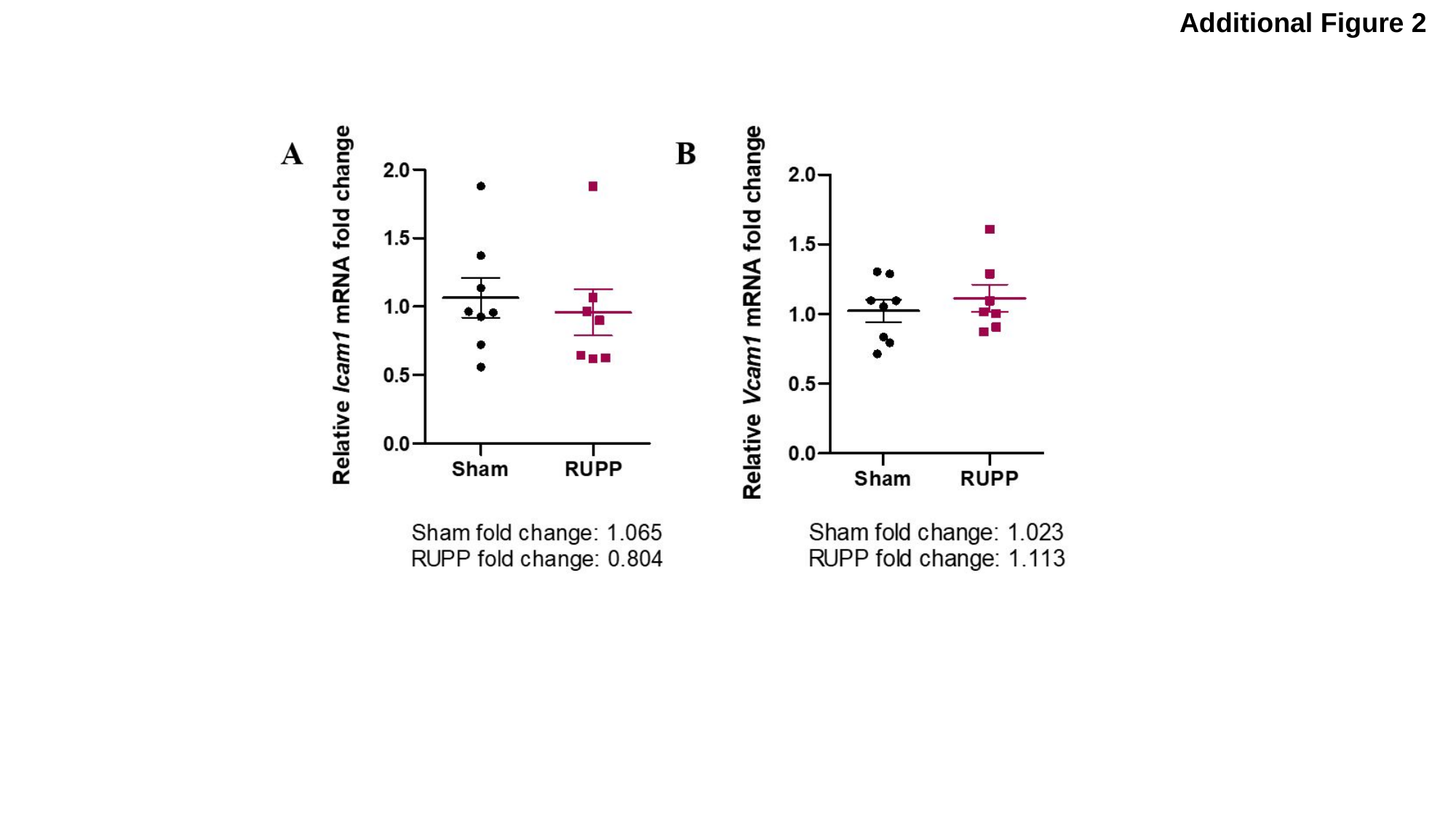

Additional Figure 2

## Slide 3
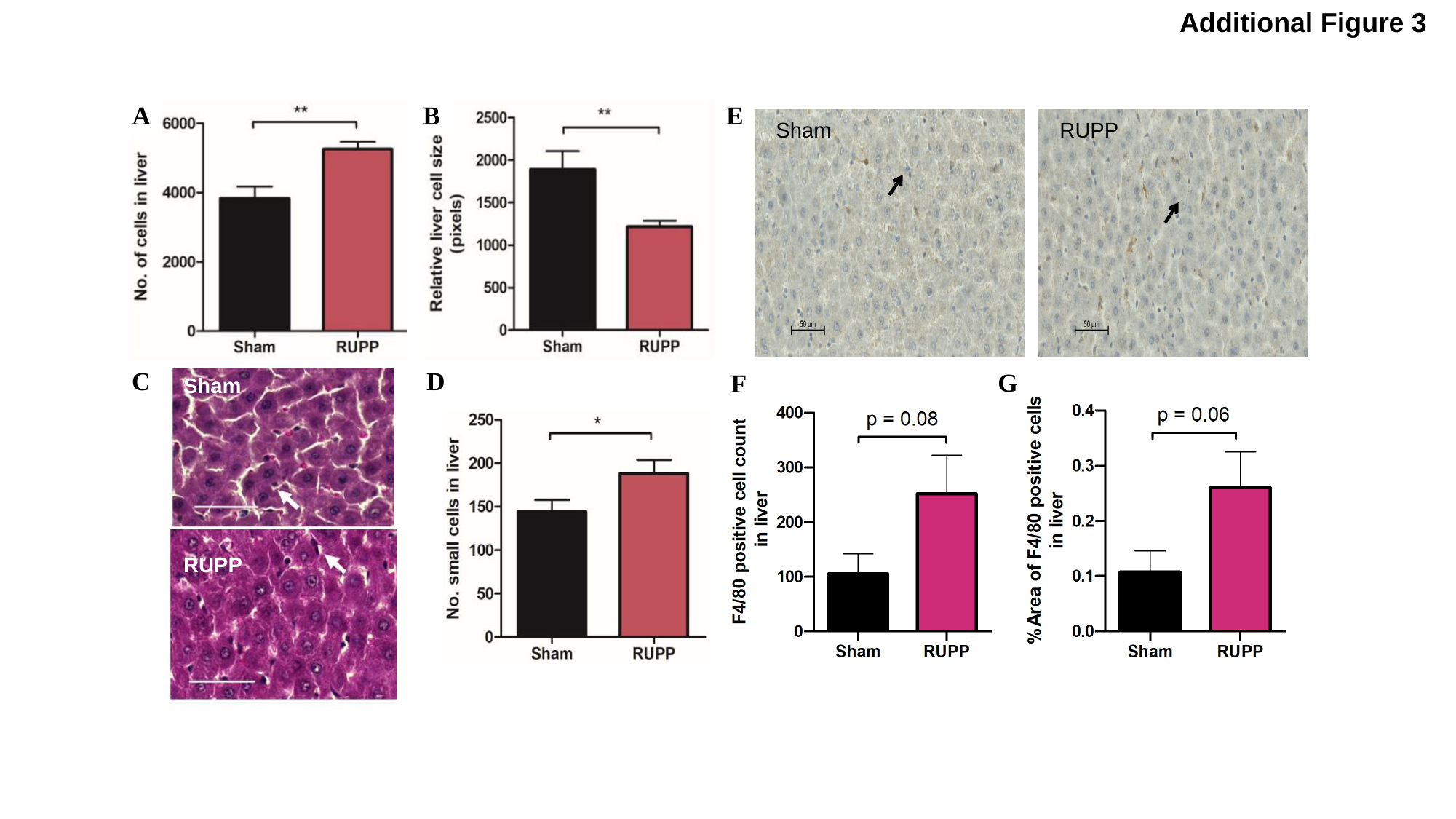

Additional Figure 3
A
B
E
Sham
RUPP
D
C
G
F
Sham
RUPP

## Slide 4
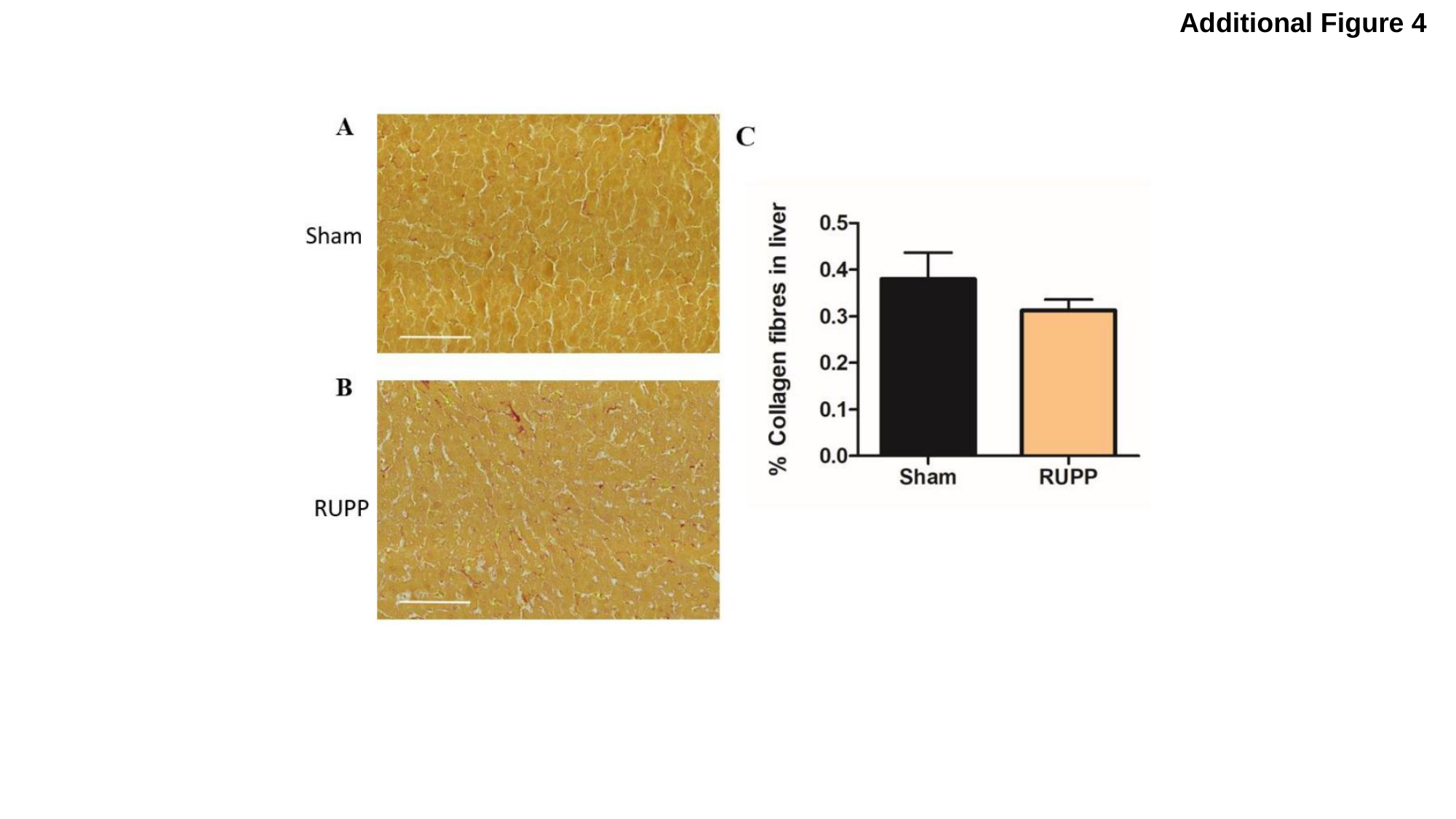

Additional Figure 4
